# Relationship between Metalloprotease-7 and -14 and Tissue Inhibitor of Metalloprotease 1 Expression by Mucosal Stromal Cells and Colorectal Cancer Development in Inflammatory Bowel Disease

**DOI:** 10.3390/biomedicines9050495

**Published:** 2021-04-30

**Authors:** Antonio Altadill, Noemi Eiro, Luis O. González, Alejandro Andicoechea, Silvia Fernández-Francos, Luis Rodrigo, José Luis García-Muñiz, Francisco J. Vizoso

**Affiliations:** 1 Department of Internal Medicine, Fundación Hospital de Jove, Avda. Eduardo Castro, 161, 33290 Gijón, Spain; altadill63@gmail.com; 2 Research Unit, Fundación Hospital de Jove, Avda. Eduardo Castro, 161, 33290 Gijón, Spain; noemi.eiro@gmail.com (N.E.); silviafernandezfrancos@gmail.com (S.F.-F.); jluisgarciamuniz@gmail.com (J.L.G.-M.); 3 Department of Anatomical Pathology, Fundación Hospital de Jove, Avda. Eduardo Castro, 161, 33290 Gijón, Spain; a.patologica2@hospitaldejove.com; 4 Department of Surgery, Fundación Hospital de Jove, Avda. Eduardo Castro, 161, 33290 Gijón, Spain; aandiko1@gmail.com; 5 Department of Gastroenterology, Central University Hospital of Asturias, Av. Roma, s/n, 33011 Oviedo, Spain; lrodigrosaez@gmail.com

**Keywords:** inflammatory bowel diseases, colorectal carcinoma, matrix metalloproteases, tissue inhibitor of metalloproteases

## Abstract

Colorectal carcinoma (CRC) associated with inflammatory bowel disease (IBD) is an example of an inflammation-related cancer. Matrix metalloproteases (MMP) are known to be associated with both processes. The aim of the study was to compare the expression of MMP-7, MMP-14 and tissue inhibitor of metalloproteases-1 (TIMP-1) in sporadic CRC- and IBD-associated CRC, and to compare the expression in inflamed and non-inflamed colonic tissue samples from IBD patients without or with associated CRC. An immunohistochemical study of MMP-7, -14 and TIMP-1 was performed on sporadic CRC (*n* = 86), IBD-associated CRC (*n* = 23) and colorectal mucosa of non-tumor samples from IBD patients without (*n* = 47) and with (*n* = 23) associated CRC. These factors were more frequently expressed by cancer-associated fibroblasts (CAF) from IBD-associated CRC than by CAF from CRC not associated with IBD. Regarding the inflamed tissue of IBD patients, Crohn’s disease (CD) patients with CRC development showed a higher expression of MMP-14 by fibroblasts and by mononuclear inflammatory cells (MICs) than CD patients without CRC development. In non-inflamed tissue samples, MMP-7 associated with fibroblasts and MICs, and TIMP-1 associated with MICs, were more frequently expressed in CD patients with CRC development than in CD patients without CRC development. Our data suggest that these factor expressions by stromal cells may be biological markers of CRC development risk in IBD patients.

## 1. Introduction

Inflammatory bowel disease (IBD) is characterized by an immune cell infiltration associated with an increased turnover of the extracellular matrix (ECM), which may result in a progressive process of architecture disruption, such as ulcer formation [1], or excessive deposition of collagen, resulting in fibrosis [2]. Matrix metalloproteases (MMP) are considered the key proteolytic enzymes responsible for the breakdown of ECM in IBD, and their high expression has also been demonstrated to be associated with the inflammation grade [3,4]. In addition, in vivo studies have shown therapeutic effects using specific MMP inhibitors [5].

Inflammation also may impact at different stages of tumor development, such as the initiation process, invasion and metastasis. Chronic inflammation promotes malignant cell transformation by triggering DNA damage or genetic instability, causing mutations and epigenetic modifications [6]. Over 25% of all cancers are related to chronic inflammation [7]. Colorectal cancer (CRC) development in IBD patients is an example of inflammation-related cancers. Chronic colonic inflammation due to IBD results in an increased risk of colon carcinogenesis, with a cumulative lifetime risk of 18–20% in ulcerative colitis (UC), and up to 8% in Crohn’s disease (CD) [8,9]. In addition, it is estimated that one out of six deaths in UC patients and one out of 12 deaths in patients with CD is caused by CRC development [8].

Among the different key signaling pathways which are up-regulated in inflammation-associated cancer, the overexpression of MMPs has attracted great interest. MMPs are also related to CRC prognosis. Several studies reported that expression of MMPs, such as MMP-1, -7, -9, -13 and -14, is significantly higher in CRC than in normal colorectal tissue [10,11], suggesting that they may have some prognostic value in CRC [12,13]. This is probably due to their known role in tumor progression through the degradation of the stromal connective tissue and basement membrane and their ability to influence the tumor cell behavior in vivo through the cleavage of growth factors, cell surface receptors, cell adhesion molecules or chemokines/cytokines [14]. Furthermore, by splitting proapoptotic factors, MMP are able to generate apoptotic-resistant cells. The activity of MMP is inhibited by specific tissue inhibitors (tissue inhibitor of metalloproteases, TIMP), which are multifactorial proteins also involved in the proliferation and the inhibition of apoptosis [15]. In fact, it has been reported that TIMP-1 and -2 are significantly higher in tumor samples [11] and associated with a poor prognosis in CRC [12].

In the present study, we analyzed the expression of MMP -7 and -14, and TIMP-1 (whose biological importance and clinical interest has been previously demonstrated in CRC [16,17] and IBD [18]), in both epithelial and stromal cells from CRC samples associated or not associated with IBD. We analyzed TIMP-1 (which inhibits MMP-7 but not MMP-14) because in a prior report we found that it was expressed differently in inflamed IBD compared to acute diverticulitis samples, unlike TIMP-2 or TIMP-3 [3]. We also analyzed the expression of these factors in IBD samples (inflamed and non-inflamed tissue) without or with CRC development.

## 2. Materials and Methods

### 2.1. Patient Selection, Patient Characteristics and Tissue Specimen Handling

Three different groups of patients were included in the present study: (1) patients with CRC not associated with IBD (*n* = 86); (2) patients with IBD-associated CRC (*n* = 23), ulcerative colitis (UC) (*n* = 13) and Crohn’s disease (CD) (*n* = 10); and (3) patients with IBD without CRC development (*n* = 47), UC patients (*n* = 16) and CD (*n* = 31). Patients with IBD-associated CRC were included whose samples and data were available in the pathological archives of the following centers: Hospital de Jove de Gijón, Hospital de Cabueñes de Gijón and Hospital Universitario Central de Asturias de Oviedo (Spain), all of them from the same geographic area, located in Asturias (Northern Region of Spain).

Clinical–pathological characteristics of patients with and without CRC are listed in Table 1 and Table 2. Samples of CRC (associated and not associated with IBD) were obtained during laparotomy (surgical procedure of tumor removal), from January 1992 to December 2005. Samples obtained from tumors, avoiding necrotic tissues, were processed for pathological examination immediately after surgical removal. Patients receiving neoadjuvant therapy were excluded. Treatment algorithms for patients with CRC were applied in a standard way according to changes along the study period.

Treatment algorithms were applied in a standard way to all patients.

Samples of IBD were obtained from 23 patients diagnosed with CRC; in 20 patients during tumor removal and in 3 patients before the CRC diagnosis.

### 2.2. Immunohistochemistry Analysis

Samples were stored as formalin-fixed, paraffin-embedded tissue blocks. Representative histological areas of tumor, inflammatory and non-inflammatory IBD tissue were defined on hematoxylin and eosin (H&E)-stained sections and marked on the slide. The counterstaining was made with hematoxylin. Tissue array (TA) [7] blocks were obtained as described previously [12]. The sections from the paraffin blocks were serially obtained and the 3 markers were incubated in sequential sections. Immunohistochemistry was performed on TA sections using a TechMate TM50 autostainer (Dako, Glostrup, Denmark), using the EnVision Detection Kit (Dako, Glostrup, Denmark). TA sections were treated in a PT-Link^®^ (Dako, Glostrup, Denmark) at 97 °C for 20 min, in citrate buffer pH 6.1 for MMP-14 (53671, AnaSpec Inc, Fremont, CA, USA) and TIMP-1 (MS-608-P0, Neomarker, Fremont, CA, USA). The dilution for each antibody was 1/100 for MMP-7 (MA5-14215, ThermoFisher, Waltham, MA, USA), 1/50 for MMP-14 and 1/100 for TIMP-1. The chromogenic substance used to demonstrate the presence of the microscopically studied protein, resulting in a brown-colored precipitate forming where the antibody bound, was diaminobenzidine (DAB). The negative control was DakoCytomation mouse serum (Dako, Glostrup, Denmark) diluted to the same mouse IgG concentration as the primary antibody. Breast tumor samples in which the presence of the evaluated proteins was confirmed by Western blot analysis were used as positive controls, as described previously [19,20]. All the dilutions were made in Antibody Diluent (Dako, Glostrup, Denmark) and incubated for 30 min at room temperature.

Factor expression was initially assessed in epithelial cells, cancer cells, fibroblast-like cells and mononuclear inflammatory cells (MICs) and classified into 2 categories; negative, ≤ 10% positive cells and positive, >10% positive cells. Nevertheless, in tissue samples positive for cells expressing either factor, at least 70% of these cells showed a positive immunostaining of each valuated field. There were at least ten 200× fields observed in each slide. Different cell types were distinguished morphologically based on their cell size; epithelial cell and tumor cells are larger than stromal cells; also, fibroblasts are spindle-shaped cells whereas MICs are small, round cells. A certified pathologist (LOG), who was blinded to the patients’ clinical results, performed the histological examination.

### 2.3. Data Analysis and Statistical Methods

The PASW Statistics 18 program was used for all calculations. Differences in percentages were calculated with the chi-square test. The specific outcomes assessed in the Data Analysis section were the comparison of factor expressions between sporadic CRC and IBD-associated CRC, and between inflamed and non-inflamed colonic tissue samples from IBD patients with or without CRC development. The results found with a *p*-value ≤ 0.05 were considered statistically significant.

## 3. Results

### 3.1. Clinical–Pathological Characteristics of Patients 

The clinical–pathological characteristics of patients with CRC development associated or not with IBD are shown in Table 1. There were no significant differences between both groups of CRC patients regarding age or gender, tumor location in the colon, histological grade or tumor stage.

No significant differences regarding clinical–pathological characteristics of IBD patients with or without CRC development (age, gender or treatment) were found, as shown in Table 2.

### 3.2. Immunohistochemical Expression of MMP-7, MMP-14 and TIMP-1

**Figure 1:**
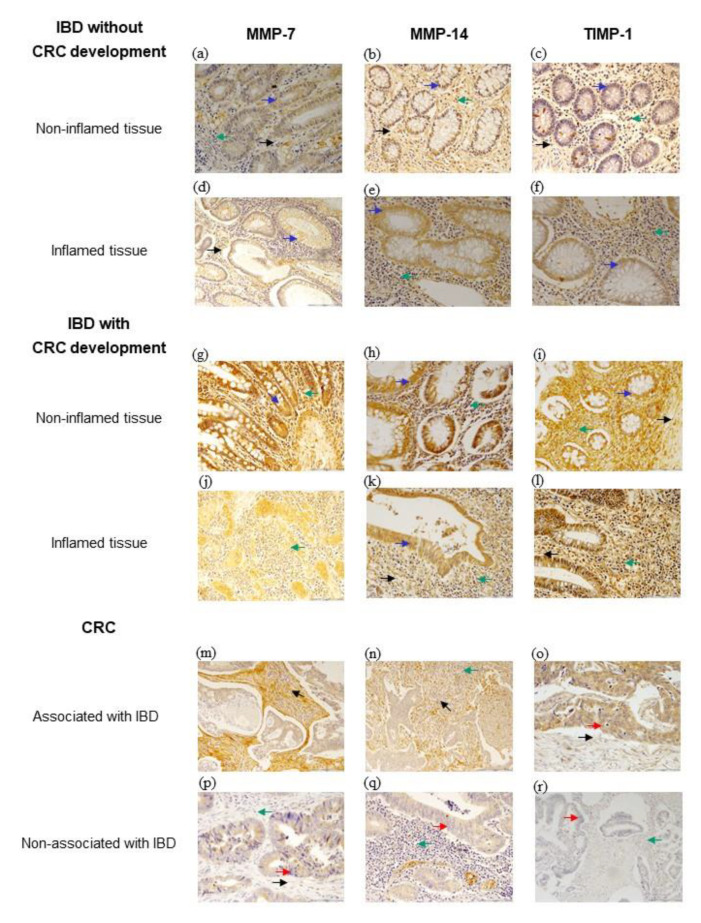
Representative examples of immunohistochemical staining for MMP-7, MMP-14 and TIMP-1 in colonic mucosa of patients depending on the group of study. 100× magnification for (**a**–**c**,**g**–**l**,**p**–**r**). 200× magnification for (**d**–**f**,**m**–**o**). The counterstaining was made with hematoxylin. Arrows indicate cell types: red, cancer cells; blue, epithelial cells; green, mononuclear inflammatory cells; and black, fibroblasts. CRC, colorectal cancer; IBD, inflammatory bowel disease. Note: Samples on tissue sections were either insufficient or lost for analysis in 10 cases for MMP-7 in CRCs not associated with IBD; in 5 cases for MMP-14 in CRCs not associated with IBD and in 8 cases of CRCs associated with IBD; in 13 cases for TIMP-1 in CRCs not associated with IBD and in 8 cases of CRCs associated with IBD.

Figure 1 shows representative examples of immunostaining for all analyzed factors in inflamed and non-inflamed IBD tissue samples without CRC development (Figure 1a–f), with CRC development (Figure 1g–l) and CRC associated (Figure 1m–o) and non-associated with IBD (Figure 1p–r). Regarding MMP-7 and TIMP-1 expression, a cytoplasmic location in positive cells was observed, whereas MMP-14 showed both cytoplasmic and membrane locations.

In CRC samples, immunostaining for MMP-7, MMP-14 and TIMP-1 were localized primarily in tumor cells, but also in stromal cells (cancer-associated fibroblasts (CAF) as well as MICs). In IBD samples, immunostaining was also localized both in epithelial cells and in stromal cells (fibroblasts and MICs).

### 3.3. Comparison of MMP-7, MMP-14 and TIMP-1 Expression of CRC Associated or Not with IBD

Table 3 shows the expressions of the studied factors in each CRC group. MMP-7 and MMP-14 were more frequently expressed by CAF from CRC associated with UC than by CAF from CRC not associated with IBD (*p* = 0.048 and *p* < 0.001, respectively). CRC from CD patients expressed more frequently MMP-14 and TIMP-1 by CAF compared with CAF from CRC not associated with IBD. However, we found no significant differences between expressions of factors by CRC from either UC or CD patients.

### 3.4. Comparison of MMP-7, MMP-14 and TIMP-1 Expression of Inflamed Tissues from IBD Patients with or without CRC Development

In UC patients, our results did not show significant differences regarding the expression of MMP-7, MMP-14 and TIMP-1 by inflamed tissues from UC patients with or without CRC development. However, our results showed that MMP-14 was more frequently expressed by fibroblasts and MICs of inflamed tissues from CD patients with CRC development than from CD patients without CRC development (*p* = 0.028 and *p* = 0.036, respectively), whereas TIMP-1 was more frequently expressed by fibroblasts from CD patients with CRC development (*p* = 0.05) (Table 4).

### 3.5. Comparison of MMP-7, MMP-14 and TIMP-1 Expression of Non-Inflamed Tissues from IBD Patients with or without CRC Development

Our results did not show significant differences between non-inflamed tissues from UC patients, with or without CRC development (Table 5). However, we found that MMP-7 and MMP-14 were more frequently expressed by fibroblasts from non-inflamed tissues of CD patients with CRC development than those of CD patients without CRC development (*p* < 0.01 and *p* < 0.05, respectively) (Table 5), whereas MMP-14 and TIMP-1 were more frequently expressed by MICs from non-inflamed tissues of CD patients with CRC development than those of CD patients without CRC development (*p* < 0.05 and *p* < 0.01, respectively). Likewise, TIMP-1 was more frequently expressed by epithelial cells from non-inflamed tissues from CD patients with CRC development than those of CD patients without CRC development (*p* < 0.05).

## 4. Discussion

**Figure 2:**
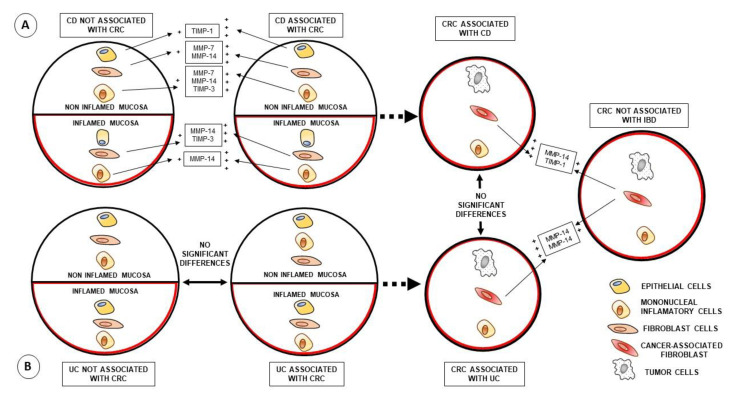
Schematic representation of the hypothesis of the evolution of the expression of metalloproteases and TIMP to cancer lesions in (**A**) Crohn’s disease (CD) and (**B**) ulcerative colitis (UC). The first column on the left shows IBD-not-associated CRC patients, and the first column on the right shows IBD-associated CRC patients (group A: CD, group B: UC). The upper part of both columns shows the highest expression of the investigated factors in patients with CD associated with CRC. Further to the right the strongest expression of the agents studied in IBD-associated CRC patients compared to CRC sporadic cases can be observed. +: expression; +++: higher expression.

The overexpression of MMP-7, MMP-14 and TIMP-1 were associated with the pathophysiology of IBD [18] and the aggressiveness of CRC [16,17]. To our knowledge, this is the first study to show that the expression of these factors is more frequent in CAF of CRC associated with IBD compared to CAF of CRCs not associated with IBD, which seems to support the hypothesis of the relationship between chronic inflammation and the development of cancer, as well as its biological aggressiveness [21,22] (Figure 2). It is known that in the context of chronic inflammation, the overexpression of pro-inflammatory cytokines, such as interleukin (IL)-1β, IL-6 and tumor necrosis factor α (TNF-α), plays a pivotal role in disease development by inducing MMP expression [23]. In addition, increased expression of *TNFR2*, one of the membrane-bound TNF receptors, was recently detected in ulcerative colitis [24]. 

It has been previously reported that the overexpression of MMP-7 in CRC correlates with more advanced Dukes stages, invasion, liver metastasis and poor outcome [25,26]. This may be due to several biological aspects of MMP-7, such as its broad substrate specificity enabling this enzyme to degrade various components of the basement membrane and extracellular matrix (for example, collagens, proteoglycans, fibronectin, laminin and elastin). MMP-7 may also contribute to cancer progression by the activation of MMP-2 and -9, and two epithelial–mesenchymal transformation pathways (RTK/RAS and the β-catenin) [27,28]. In addition, MMP-7 may promote angiogenesis through a direct or indirect proliferative effect on endothelial cells, enriching the variety of angiogenesis mediators and secreting angiogenesis inhibitors [29,30]. Interestingly, it has been also suggested that MMP-7 can contribute to the expansion of tumor stroma, stimulating the proliferation and migration of human colonic fibroblasts [31]. In this sense, it has been widely described that CAF contribute to a wide range of pro-tumor actions [32]. A recent study showed that FGF-1/-3/FGFR4 signaling in CAF promotes tumor progression in colon cancer through ERK and MMP-7, inducing angiogenesis and cell proliferation [33], which suggests a crucial role of CAF and FGF signaling in colorectal cancer. MMP-14 also contributes to the degradation of the extracellular matrix by activating other MMPs, such as pro-MMP-13 and pro-MMP-2, and plays a crucial role in molecular carcinogenesis and tumor growth. All of these functions might explain the association of MMP-14 expression with poorer prognosis in CRC patients [34]. Taken together, our data suggest that MMP-7 and -14 expression may contribute to identify a phenotype of CAF, associated with CRC development in IBD patients.

Considering that one of the major sources of CAF are resident fibroblasts, it seems reasonable to investigate fibroblasts of colonic mucous from IBD patients with and without CRC development. Figure 1 outlines the evolutionary hypothesis that we considered based on our results. Our data led us to contemplate the existence of one stromal cell phenotype in both non-inflammatory and inflammatory tissues prior to cancer development, in one significant percentage of patients with CD. Our data also show an increased TIMP-1 expression by MICs from non-inflamed tissue samples from CD patients with CRC development, compared to patients without CRC development. TIMP-1 can promote cell growth and inhibit apoptosis [35]. In addition, there are data indicating TIMP-1 might play a role in colorectal carcinogenesis from colorectal adenomas [17]. Especially relevant was our finding of a phenotype of fibroblasts expressing MMP-7 and MMP-14 in benign non-inflamed colorectal tissues associated with further CRC in CD patients. This suggests that both MMPs might be possible molecular markers of stromal cells from CD patients predisposed to CRC. In this sense, in adenomatous polyposis coli (APC), an increased expression of MMP-7 may serve as an early indicator for colorectal carcinogenesis [18], which may be related to the fact that MMP-7 activation is directly associated with APC mutations [36]. On the contrary, it has been reported that mice lacking MMP-7 expression have a reduced incidence of colonic adenoma [37]. In accordance with all of these observations, a recent bioinformatic analysis study based on micro array databases (GSE10950, GSE44861 and GSE744602) identified MMP-7 as the more important gene expression involved in colon carcinogenesis, invasion and recurrence of colon cancer [38]. This is consistent with the fact that MMP-7 was reported to regulate the occurrence and development of cancer and mediate the proliferation, differentiation, metastasis and invasion of diverse types of cancer cells through various mechanisms (revised by [39]).

Identifying at-risk patients and carrying out appropriate preventive measures such as chemoprophylaxis or periodic colonoscopy surveillance of these patients is of great importance as a clinical strategy to lessen the hazard of CDC in IBD patients. Several clinical factors have been associated with an increase in risk, such as young age at the onset of IBD, major disease extension, longer duration of disease, primary sclerosing cholangitis, degree of inflammation or the presence of moderate to high dysplasia [40]. At present, surveillance is especially recommended for patients with long-term IBD. However, the fact that up to 30% of CRC cases were interval cancers (that appeared after a negative screening test or examination) indicates the difficult management of this issue and the need to identify the most precise factors for CRC [41]. Therefore, there is today the need to have more precise markers that help to achieve a better discrimination of IBD subtypes that jeopardize CRC development, as well as the need to identify proteomic biomarkers to diagnose or predict cancer risk.

Obviously, the size of the sample and the retrospective design are the key limitations of the present study. In our hospital records, we encountered great difficulty in recruiting well-documented cases, as well as optimal tissue samples, for the purpose of deepening the relationship between IBD and CRC development. This probably reflects a deficient sensitivity in our clinical setting to assess this important connection in the context of the paradigm of the relationship between inflammation and cancer. Therefore, the reported outcomes should be considered as preliminary and deserve to be validated. In addition, it will be important to determine whether they find similar expression patterns in pre-cancerous stages of IBD.

To summarize, we believe our findings seem to support the hypothesis of the relationship between intestinal inflammation and cancer. In addition, they led us to consider that further studies on the correlation between MMP-7 and MMP-14 and their inhibitors TIMP-1 and TIMP-2, respectively, based on double staining, as well as eventually others (of proliferation and/or apoptosis) and also of MMP-14 with TIMP-2, may be of interest in the context of the relationship between IBD and CRC.

## Figures and Tables

**Table 1:** Basal characteristics of 109 patients with colorectal cancer.

Characteristics	CRCs Not Associated with IBD N (%)	CRCs Associated with IBD N (%)	*p*-Value
**All patients**	86 (100)	23 (100)	
**Age (years)**			0.070
≤64	43 (50)	17 (73.9)	
>64	43 (50)	6 (26.1)	
**Gender**			0.922
Male	49 (56.9)	14 (60.9)	
Female	37 (43)	9 (39.1)	
**Tumor location**			0.399
Right	13 (15.1)	5 (21.7)	
Transverse	2 (2.3)	2 (8.7)	
Left	34 (39.5)	7 (30.4)	
Rectum	37 (43)	9 (39.1)	
**Histological grade**			0.438
Well differentiated (I)	16 (18.6)	5 (21.7)	
Moderately differentiated (II)	65 (75.5)	15 (65.2)	
Poorly differentiated (III)	5 (5.8)	3 (13.0)	
**Duke’s stage**			0.869
A	6 (6.9)	1 (4.8)	
B	49 (56.9)	12 (50.5)	
C	17 (19.7)	6 (27.3)	
D	14 (16.3)	3 (13.0)	

Abbreviations: CRC, colorectal cancer; IBD, inflammatory bowel disease. Dukes’ stage unknown for 1 case of CRC associated with IBD.

**Table 2:** Clinical characteristics of IBD patients at the time of the study inclusion.

Clinical Characteristics	IBD Patients without CRCs Development N (%)	IBD Patients with CRCs Development N (%)	*p*-Value
**All patients**	47 (100)	23 (100)	
**Age (years)**			0.615
≤38	27 (57.4)	11 (47.8)	
>38	20 (42.6)	12 (52.2)	
**Gender**			1
Male	28 (59.6)	14 (60.9)	
Female	19 (40.4)	9 (39.1)	
**Immunosuppressive treatment**			0.304
No	29 (67.4)	19 (82.6)	
Yes	14 (32.6)	4 (17.4)	
**All patients**	47 (100)	23 (100)	
**Age (years)**			0.615
≤38	27 (57.4)	11 (47.8)	
>38	20 (42.6)	12 (52.2)	
**Gender**			1
Male	28 (59.6)	14 (60.9)	
Female	19 (40.4)	9 (39.1)	
**Immunosuppressive treatment**			0.304
No	29 (67.4)	19 (82.6)	
Yes	14 (32.6)	4 (17.4)	
**All patients**	47 (100)	23 (100)	

Abbreviations: CRC, colorectal cancer; IBD, inflammatory bowel disease. Note: It was impossible to know if they were taking immunosuppressant medication at the time of surgery in 4 cases of CD without CRC development.

**Table 3:** Expression of MMP-7, MMP-14 and TIMP-1 by different cell types in CRCs not associated and associated with IBD (% of cases refers to the total number of cases analyzed indicated for each protein).

Factors	CRCs Not Associated with IBD N (%)	CRCs Associated with CD N (%)	CRC Associated with UC N (%)
**MMP-7**	86 (100)	9 (100)	13 (100)
Tumor cells	67 (77.9)	7 (77.8)	1 (7.7)
Fibroblasts	33 (38.4)	5 (55.6)	9 (69.2) *
MICs	19 (22.1)	3 (33.3)	5 (38.5)
**MMP-14**	86 (100)	6 (100)	8 (100)
Tumor cells	71 (82.6)	5 (83.3)	6 (75)
Fibroblasts	13 (15.1)	4 (66.7) **	6 (75) ***
MICs	5 (5.8)	1 (16.7)	1 (12.5)
**TIMP-1**	86 (100)	6 (100)	8 (100)
Tumor cells	69 (80.2)	6 (100)	0 (0)
Fibroblasts	16 (18.6)	4 (66.7) **	2 (25)
MICs	27 (31.4)	1 (16.7)	1(12.5)

Abbreviations: CD, Chron’s disease; CRC, colorectal cancer; IBD, inflammatory bowel disease; MIC, mononuclear inflammatory cell; UC, ulcerative colitis. * *p* < 0.05 CRC without IBD development vs. IBD (UC) with CRC development. ** *p* < 0.05 CRC without IBD development vs. IBD (CD) with CRC development. *** *p* < 0.01 CRC without IBD development vs. IBD (UC) with CRC development.

**Table 4:** Expression of MMP-7, MMP-14 and TIMP-1 by different cell types in inflamed tissue in IBD without or with CRC development (% of cases refers to the total number of cases analyzed indicated for each protein).

Factors	IBD without CRC Development N (%)		IBD with CRC Development N (%)	
	CD	UC	CD	UC
**MMP-7**	29 (100)	16 (100)	8 (100)	7 (100)
Epithelial cells	22 (75.9)	9 (56.3)	6 (75)	4 (57.1)
Fibroblasts	13 (44.8)	7 (43.8)	5 (62.5)	3 (42.9)
MICs	15 (51.7)	3 (18.8)	5 (62.5)	3 (42.9)
**MMP-14**	26 (100)	5 (100)	7 (100)	4 (100)
Epithelial cells	12 (46.2)	4 (80)	5 (71.4)	3 (75)
Fibroblasts	5 (19.2)	4 (80)	5 (71.4) *	2 (50)
MICs	3 (11.5)	3 (60)	4 (57.1) *	0 (0)
**TIMP-1**	27 (100)	15 (100)	7 (100)	5 (100)
Epithelial cells	10 (37)	7 (46.7)	3 (42.9)	1 (20)
Fibroblasts	0	2 (13.3)	2 (28.6) **	0
MICs	3(11.1)	3 (20)	0	0

Abbreviations: CD, Crohn´s Disease; CRC, colorectal cancer; IBD, inflammatory bowel disease; MICs, mononuclear inflammatory cells; UC, ulcerative colitis. * *p* < 0.05 IBD (CD) without CRC development vs. IBD (CD) with CRC development. ** *p* ≤ 0.05 IBD (CD) without CRC development vs. IBD (CD) with CRC development. Note: Samples on tissue sections were either insufficient or lost for analysis in some cases, as indicated for each protein.

**Table 5:** Expression of MMP-7, MMP-14 and TIMP-1 by different cell types in non-inflamed tissue in IBD without or with CRC development. (% of cases refers to the total number of cases analyzed indicated for each protein).

Factors	IBD without CRC Development N (%)		IBD with CRC Development N (%)	
	CD	UC	CD	UC
**MMP-7**	31 (100)	16 (100)	9 (100)	11 (100)
Epithelial cells	17 (54.8)	12 (75)	7 (77.8)	10 (90.9)
Fibroblasts	4 (12.9)	12 (75)	6 (66.7) **	10 (90.9)
MICs	5 (16.1)	6 (37.5)	5 (55.6) *	9 (81.8)
**MMP-14**	27 (100)	15 (100)	6 (100)	8 (100)
Epithelial cells	6 (22.2)	9 (60)	4 (66.7)	1 (12.5)
Fibroblasts	1 (3.7)	8 (53.3)	3 (50) *	1 (12.5)
MICs	0	2 (13.3)	2 (33.3) *	0
**TIMP-1**	27 (100)	15 (100)	6 (100)	8 (100)
Epithelial cells	10 (37)	9 (60)	6 (100) *	1 (12.5)
Fibroblasts	1 (3.7)	2 (13.3)	1 (16.7)	0
MICs	2 (7.4)	0	4 (66.7) **	0

Abbreviations: CD, Crohn’s Disease; CRC, colorectal cancer; IBD, inflammatory bowel disease; MICs, mononuclear inflammatory cells; UC, ulcerative colitis. * *p* < 0.05 IBD (CD) without CRC development vs. IBD (CD) with CRC development. ** *p* < 0.01 IBD (CD) without CRC development vs. IBD (CD) with CRC development. Note: Samples on tissue sections were either insufficient or lost for analysis in some cases, as indicated for each protein).

## Data Availability

The datasets generated and/or analyzed during the current study are available from the corresponding author upon reasonable request.
